# Human milk phospholipids across lactation stages and their associations with infant neurodevelopment: a prospective cohort study in China

**DOI:** 10.3389/fnut.2026.1841752

**Published:** 2026-05-21

**Authors:** Sihan Yang, Qianying Guo, Tianyang Zheng, Nan Yang, Chen Yang, Mingxuan Cui, Tianmai Li, Xinran Liu, Hai Lin, Qianhui Cheng, Sainan Li, Peng Liu, Linlin Wang

**Affiliations:** 1 Institute of Reproductive and Child Health/National Health Commission Key Laboratory of Reproductive Health, Department of Epidemiology and Biostatistics, School of Public Health, Peking University, Beijing, China; 2 Department of Toxicology, Institute of Reproductive and Child Health/National Health Commission Key Laboratory of Reproductive Health, School of Public Health, Peking University, Beijing, China; 3 State Key Laboratory of Female Fertility Promotion, Peking University, Beijing, China; 4 Department of Clinical Nutrition, Peking University People's Hospital, Beijing, China; 5 College of Science and Medicine, Australia National University, Canberra, ACT, Australia; 6 Beijing Key Laboratory of Toxicological Research and Risk Assessment for Food Safety, Beijing, China

**Keywords:** Chinese human milk, elastic net, infant neurodevelopment, lactation stages, phospholipids

## Abstract

**Introduction:**

Early-life nutrition is a key determinant of infant neurodevelopment. Phospholipids (PLs) in human milk contribute to neuronal membrane structure and signaling, yet population-based evidence linking milk PLs to early neurodevelopment remains limited. This study aimed to examine dynamic changes in breast milk PLs across lactation and their associations with infant neurodevelopment at 6 months.

**Methods:**

In this prospective cohort study, 50 mother–infant dyads were recruited at Peking University People’s Hospital. Human milk samples were collected at four lactation stages: colostrum (2 days), transitional milk (15 days), 1-month mature milk, and 6-month mature milk. PL concentrations were quantified using UPLC–MS/MS. Infant neurodevelopment at 6 months was assessed using the Ages and Stages Questionnaires (ASQ-3). Associations between milk PLs and neurodevelopment were analyzed using multivariable linear regression, with Elastic Net regression and bootstrap resampling applied to identify PLs most stably associated with neurodevelopment. Sensitivity analyses including additional PLs (PS and PA) were conducted to evaluate the robustness of findings.

**Results:**

A total of 148 PL species across 13 classes were quantified. Total PL decreased from colostrum to mature milk, with distinct patterns observed for individual subclasses. In multivariable regression, higher LPC and Cer in colostrum and PI in 1-month mature milk were positively associated with ASQ-3 scores. Elastic Net analysis highlighted several PLs in colostrum with relatively higher selection probabilities. Joint modeling revealed that Cer, LPC, and PC were positively associated with neurodevelopment, whereas PE was negatively associated.

**Conclusion:**

Breast milk PLs exhibit marked temporal variations across lactation, and early-lactation PLs, particularly in colostrum, are associated with infant neurodevelopment at 6 months. These findings underscore the potential importance of colostrum PLs in early brain development and highlight the need for further studies with larger cohorts, longer follow-up, and molecular-level lipidomic analyses.

## 1 Introduction

Infancy represents a critical period for human neurodevelopment. During this stage, the brain undergoes rapid neuronal proliferation, synaptogenesis, and neural network remodeling, which play essential roles in shaping later cognitive, language, motor, and socio-emotional functions (1, 2). Previous studies have shown that infant neurodevelopment is influenced by multiple factors, including genetic, environmental, and nutritional determinants. Among these, early-life nutrition has been recognized as an important modifiable factor that may promote optimal brain development (3–5).

Human milk is widely regarded as the optimal source of nutrition for infants, particularly during the first 6 months of life. It contains a wide range of nutrients and bioactive components that support infant growth and neurodevelopment. Phospholipids (PLs), as important structural components of cell membranes, contribute to neuronal membrane stability and are involved in multiple neurobiological processes, including myelination, synaptic signal transmission, and neuronal proliferation (6–9). Therefore, PLs are considered to play a crucial role in the development of the infant nervous system. In human milk, PLs are primarily located within the milk fat globule membrane (MFGM) and comprise several subclasses, including phosphatidic acid (PA), phosphatidylcholine (PC), phosphatidylethanolamine (PE), phosphatidylglycerol (PG), phosphatidylinositol (PI), phosphatidylserine (PS), and sphingomyelin (SM) (10). Although PLs account for only approximately 0.2–2.0% of total milk lipids, they are considered important contributors to infant brain development (11).

Evidence from both animal experiments and clinical studies supports the potential role of PLs in neurodevelopment. Animal studies have shown that phospholipid-enriched milk can enhance neural plasticity, promote brain development, and improve cognitive performance in young animals (12–15). In addition, randomized controlled trials have reported that supplementation of infant formula with milk fat globule membrane components may promote brain myelination and improve certain cognitive and motor developmental outcomes in infants (16–19).

Despite these findings, population-based evidence linking human milk PLs with infant neurodevelopment remains limited. To date, most human studies have focused on describing changes in human milk phospholipid composition across lactation, whereas studies investigating their associations with infant neurodevelopment remain limited (20–22). The first epidemiological evidence connecting human milk PLs with early children cognitive development was found in a study from the MUAI cohort (23). However, rather than focusing on neurodevelopment during infancy, the study primarily examined cognitive results in preschool-aged children. Moreover, phospholipid subclasses are often highly correlated, and analyses based on single components may not adequately capture overall phospholipid exposure in human milk. These considerations highlight the need to investigate the associations between human milk PLs and infant neurodevelopment from an integrated lipidomic perspective.

In this prospective mother–infant cohort study, we collected human milk samples at different lactation stages and quantified phospholipid concentrations, while assessing infant neurodevelopment at 6 months of age. We aimed to examine the associations between human milk phospholipids across lactation and early neurodevelopment in infants, and to provide epidemiological evidence to improve understanding of the potential role of human milk PLs in infant neurodevelopment.

## 2 Materials and methods

### 2.1 Study population

This study employed a prospective cohort design. Participants were women who received prenatal care and delivered at Peking University People’s Hospital between October 2020 and October 2023, along with their full-term infants (gestational age ≥37 weeks). Eligible participants were aged 20–45 years and agreed to provide human milk samples and complete questionnaires during follow-up. Participants were excluded if they did not provide informed consent, were lost to follow-up, had severe hepatic or renal diseases or major psychiatric disorders, or had infectious diseases (e.g., HIV, hepatitis B). A total of 50 mother-infant dyads were ultimately included in the study. This study was approved by the Ethics Committee of Peking University People’s Hospital, and written informed consent was obtained from all participants.

### 2.2 Data collection

Trained investigators collected baseline maternal information through face-to-face interviews at enrollment, including maternal age, height, pre-pregnancy weight, pre-pregnancy BMI, parity, date of delivery, mode of delivery, and smoking status.

At the 6-month follow-up, infant feeding information was collected using a structured questionnaire, including feeding mode (exclusive or mixed), feeding frequency (times/day), feeding interval (hours), and volume per feeding (mL). Daily human milk intake was estimated based on feeding frequency and volume per feeding.

### 2.3 Human milk sample collection, processing, and PLs analysis

Human milk samples were collected at four lactation stages: colostrum (C, 2 days postpartum), transitional milk (T, 15 days postpartum), 1-month mature milk (M1, 1 month postpartum), and 6-month mature milk (M6, 6 month postpartum). Sample collection was standardized to the second morning feeding (between 9:00 and 11:00 a.m.). Prior to collection, mothers cleaned the breast area. Human milk was fully expressed from one breast manually and collected into sterile milk storage bags. After collection, samples were temporarily stored at −20 °C and either transported to the hospital by participants or collected by researchers. Upon arrival at the laboratory, samples were homogenized, aliquoted into cryovials, and immediately stored at −80 °C until analysis.

PLs were quantified using ultra-performance liquid chromatography coupled with tandem mass spectrometry (UPLC-MS/MS, ExionLTM LC AD system, QTRAP® 6,500+) with an electrospray ionization (ESI) source. Chromatographic separation was performed on a Thermo AccucoreTM C30 column (2.6 μm, 2.1 mm × 100 mm) maintained at 45 °C. Gradient elution was conducted with mobile phase A (acetonitrile/water, 60:40, v/v, 0.1% formic acid, 10 mmol/L ammonium formate) and mobile phase B (acetonitrile/isopropanol, 10:90, v/v, 0.1% formic acid, 10 mmol/L ammonium formate) at 0.35 mL/min.

Phospholipid identification was performed in multiple reaction monitoring (MRM) mode based on characteristic precursor-to-product ion transitions and confirmed using an in-house lipid reference library (Metware Biotechnology Co., Ltd., Wuhan, China). Internal standards provided by the analytical platform were added to each sample prior to lipid extraction. Peak detection and integration were conducted from chromatographic data. Phospholipid concentrations were quantified using an internal standard–based approach, and lipid abundances were calculated from the peak area ratios of analytes to internal standards, together with known standard concentrations and correction factors.

Quality control was ensured using pooled QC samples injected at regular intervals (every 10 samples) throughout the analytical run. Instrument stability and analytical reproducibility were evaluated by total ion chromatogram (TIC) overlap, QC sample correlation analysis, and coefficient of variation (CV) assessment, demonstrating stable instrument performance and high data repeatability.

### 2.4 Infant neurodevelopment assessment

At the 6-month follow-up, infant neurodevelopment was assessed via caregiver interviews by trained investigators using the Ages and Stages Questionnaires®, Third Edition (ASQ-3). The ASQ-3 evaluates five developmental domains: communication, gross motor, fine motor, problem solving, and personal-social. Domain scores were summed to calculate the ASQ-3 total score, reflecting overall neurodevelopment. Higher scores indicate better neurodevelopment.

### 2.5 Statistical analysis

Categorical variables are presented as frequencies and percentages. Phospholipid concentrations, which were not normally distributed, are presented as median (interquartile range, IQR). PL concentrations across the four lactation stages were compared using the Friedman test. Following a significant result (*p* < 0.05), post-hoc pairwise comparisons were performed using the Nemenyi test to identify specific stage-to-stage differences.

Associations between human milk PLs and infant neurodevelopment at 6 months were examined using multiple linear regression models for each lactation stage, adjusting for maternal age, pre-pregnancy BMI, parity, mode of delivery, feeding mode, passive smoking, and infant sex.

To account for potential correlations among phospholipid subclasses, Spearman correlation analysis was performed. Elastic Net regression, which combines LASSO and Ridge penalties to improve model stability in the presence of correlated variables, was used to identify PLs most strongly associated with infant neurodevelopment. Separate Elastic Net models were constructed for each lactation stage using a bootstrap resampling procedure with 1,000 iterations, in each iteration, 80% of samples were randomly selected. Within each iteration, the optimal penalty parameter (*λ*) was determined using 10-fold cross-validation, and models were fitted with *α* = 0.5. Selection probabilities were calculated as the proportion of times each variable was retained across all iterations. Phospholipids with relatively higher and stable selection probabilities were identified based on the distribution of selection frequencies, while variables with consistently low selection probabilities were excluded. The selected phospholipids were then included in multivariable linear regression models to evaluate their joint associations with infant neurodevelopment. To assess the robustness of variable selection, sensitivity analyses were conducted using more inclusive sets of phospholipids. Multicollinearity among selected variables was evaluated using the variance inflation factor (VIF).

All analyses were conducted using R 4.5.0. Two-sided *p* values <0.05 were considered statistically significant.

## 3 Results

### 3.1 Participants

A total of 50 mother-infant dyads were enrolled, and human milk samples were collected at four lactation stages. After excluding four participants lost to follow-up, 46 infants had neurodevelopmental assessments at 6 months.

The median maternal age was 31 years (IQR: 29–34), with 42.0% of mothers aged 30–34 years. Most mothers had a normal pre-pregnancy BMI (72%), 18% were overweight or obese, and 10% were underweight. Primiparous women accounted for 66% of the cohort. Delivery occurred vaginally in 68% of mothers and by cesarean section in 32%. Infant feeding included exclusive breastfeeding in 44% and mixed feeding in 56% of infants. The majority of mothers (80%) reported no passive smoking exposure during pregnancy or postpartum. Among the 46 infants, 20 (43.5%) were male and 26 (56.5%) were female. Detailed demographic and clinical characteristics are summarized in Table 1.

**Table 1:** Characteristics of study participants.

Characteristics	n (%)
Maternal age (years)	
25–29	18 (36.0)
30–34	21 (42.0)
≥35	11 (22.0)
Pre-pregnancy BMI (kg/m^2^)	
<18.5	5 (10.0)
18.5–23.9	36 (72.0)
≥24	9 (18.0)
Parity	
Primiparous	33 (66.0)
Multiparous	17 (34.0)
Mode of delivery	
Vaginal	34 (68.0)
Cesarean	16 (32.0)
Passive smoking	
None	40 (80.0)
Less than once a week	3 (6.0)
Once a week or more	7 (14.0)
Infant feeding mode	
Exclusive breastfeeding	22 (44.0)
Mixed feeding	28 (56.0)
Infant sex (*n* = 46)	
Boy	20 (43.5)
Girl	26 (56.5)

### 3.2 Human milk PL concentrations and longitudinal changes over lactation

A total of 148 PL species were detected in 200 human milk samples and classified into 13 subclasses. Lysophosphatidic acid (LPA) was detected in less than 50% of samples and was therefore excluded from further analysis. Among the remaining subclasses, SM, PA, and PC were the most abundant.

**Figure 1:**
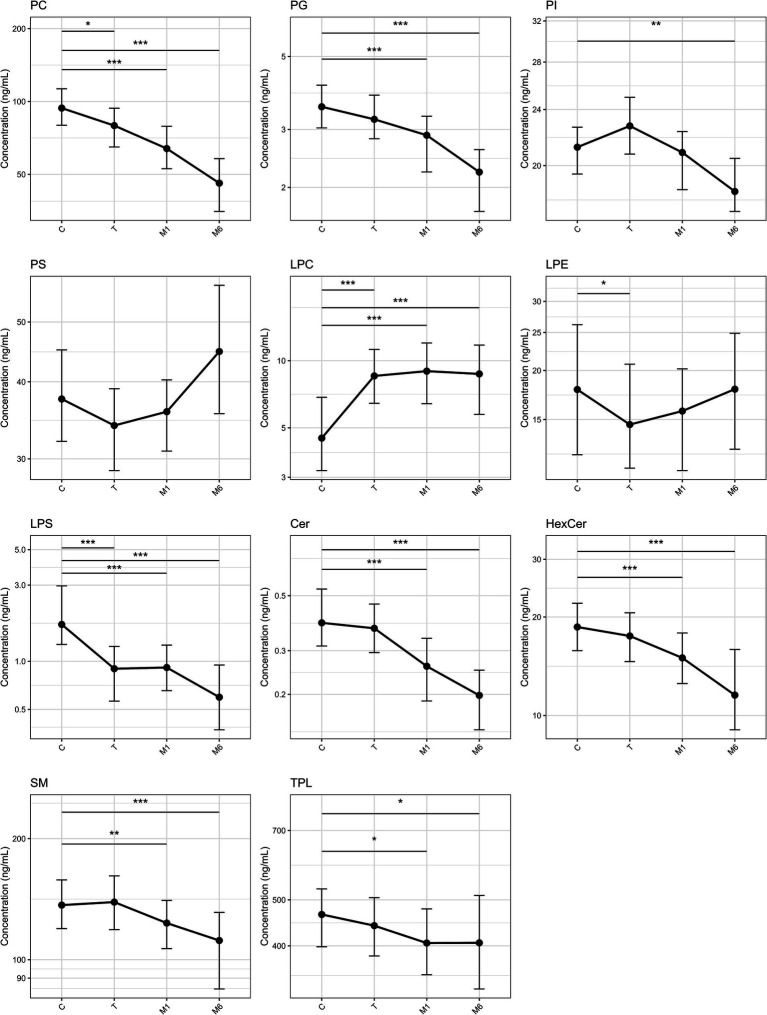
Longitudinal changes of breast milk phospholipid concentrations across lactation stages. Lines represent median concentrations and error bars indicate interquartile ranges (IQR). The y-axis is presented on a logarithmic scale. Phospholipids showing significant overall differences by Friedman test are presented. *p < 0.05, **p < 0.01, ***p < 0.001 for pairwise comparisons between colostrum and each later stage (C vs. T, C vs. M1, C vs. M6) using the Nemenyi post-hoc test. C, colostrum; T, transitional milk; M1, 1-month mature milk; M6, 6-month mature milk; Cer, ceramide; HexCer, hexosylceramide; LPC, lysophosphatidylcholine; LPE, lysophosphatidylethanolamine; LPS, lysophosphatidylserine; PC, phosphatidylcholine; PG, phosphatidylglycerol; PI, phosphatidylinositol; PLs, phospholipids; PS, phosphatidylserine; SM, sphingomyelin; TPL, total phospholipid.

Overall, total phospholipid (TPL) concentrations decreased from C to M1 and then remained stable, with a significant difference between C and M1 (*p* < 0.001), while no difference was observed between M1 and M6. Among the six glycerophospholipids (GPLs), PA and PC had relatively higher concentrations. PC and PG concentrations declined continuously from C to M6 (*p* < 0.001), whereas PA and PE showed no significant changes over lactation. PI exhibited a non-linear trajectory, increasing from C to T and subsequently decreasing, with significantly lower levels in M6 compared with both T and C (T vs. M6, *p* < 0.001; C vs. M6, *p* = 0.002). PS showed a U-shaped trajectory, with significantly higher levels at M6 compared with T (*p* < 0.001). Among the three lysophospholipids (Lyso-PLs), lysophosphatidylethanolamine (LPE) exhibited relatively higher concentrations and exhibited relatively minor variation, with only a modest difference between T and C (*p* = 0.034), while other comparisons were not significant. Lysophosphatidylcholine (LPC) increased from C to T (*p* < 0.001) and remained stable thereafter, with no differences observed among later stages. In contrast, lysophosphatidylserine (LPS) showed a progressive decline across lactation, with significantly lower levels in M1 and M6 compared with C (both *p* < 0.001). For the three sphingolipids, SM was the most abundant. Ceramide (Cer), hexosylceramide (HexCer), and SM all decreased over lactation, with significantly lower levels in both M1 and M6 compared with C (all *p* < 0.001). Detailed results are presented in Table 2, Figure 1, and Supplementary Table S1.

**Table 2:** Breast milk PL subclass concentrations across lactation stages (*n* = 50).

PLs	C	T	M1	M6
GPLs				
PA	96.07 (80.53–108.64)	104.49 (87.77–118.89)	100.89 (85.43–134.01)	105.89 (67.54–183.90)
PC^3^	93.92 (79.73–112.90)	79.52 (64.87–93.79)	63.91 (52.73–78.99)	45.99 (35.15–58.08)
PE	15.44 (12.76–18.26)	16.79 (12.50–19.29)	14.14 (10.62–16.06)	14.86 (11.04–18.78)
PG^3^	3.52 (3.03–4.09)	3.22 (2.81–3.81)	2.88 (2.23–3.29)	2.23 (1.69–2.61)
PI^3^	21.23 (19.46–22.66)	22.75 (20.76–24.97)	20.87 (18.49–22.34)	18.39 (17.23–20.48)
PS^3^	37.51 (32.02–45.06)	33.98 (28.71–38.97)	35.77 (30.88–40.29)	44.78 (35.51–57.34)
Lyso-PLs				
LPC^3^	4.49 (3.21–6.85)	8.55 (6.44–11.24)	8.98 (6.42–12.01)	8.72 (5.74–11.75)
LPE^1^	17.89 (12.20–26.17)	14.56 (11.28–20.76)	15.76 (11.12–20.19)	17.93 (12.60–24.89)
LPS^3^	1.70 (1.28–2.97)	0.90 (0.56–1.24)	0.91 (0.66–1.26)	0.60 (0.37–0.95)
Sphingolipids				
Cer^3^	0.39 (0.31–0.53)	0.37 (0.29–0.46)	0.26 (0.19–0.34)	0.20 (0.14–0.25)
HexCer^3^	18.64 (15.78–22.02)	17.50 (14.61–20.60)	15.00 (12.52–17.88)	11.54 (9.05–15.92)
SM^3^	136.70 (119.46–157.93)	139.08 (118.78–161.60)	123.34 (106.57–140.39)	111.62 (84.57–131.09)
TPL^1^	465.78 (398.21–527.28)	441.11 (380.79–505.37)	405.46 (347.70–478.46)	405.90 (324.49–510.60)

Data are presented as median (IQR), ng/mL. ^1^P < 0.05, ^3^P < 0.001 as determined using the Friedman test for differences across lactation stages. Cer, ceramide; GPLs, glycerophospholipids; HexCer, hexosylceramide; LPC, lysophosphatidylcholine; LPE, lysophosphatidylethanolamine; LPS, lysophosphatidylserine; Lyso-PLs, lysophospholipids; PA, phosphatidic acid; PC, phosphatidylcholine; PE, phosphatidylethanolamine; PG, phosphatidylglycerol; PI, phosphatidylinositol; PLs, phospholipids; PS, phosphatidylserine; SM, sphingomyelin; TPL, total phospholipid.

### 3.3 Associations between human milk PLs and infant neurodevelopment across lactation stages

Multivariable linear regression models were applied for each lactation stage, adjusting for maternal age, pre-pregnancy BMI, parity, mode of delivery, feeding mode, passive smoking, and infant sex. In C, higher concentrations of LPC and Cer were significantly associated with higher neurodevelopment scores at 6 months (LPC: *β* = 9.93, *p* = 0.017; Cer: *β* = 8.24, *p* = 0.047). In M1, PI showed a significant positive association with neurodevelopment (*β* = 8.86, *p* = 0.041). No statistically significant associations were observed for T or M6. Detailed results are presented in Table 3.

**Table 3:** Associations between human milk PLs and infant neurodevelopment at different lactation stages: multivariable linear regression models (*n* = 46).

PLs	C	T	M1	M6
	β (95%CI)	β (95%CI)	β (95%CI)	β (95%CI)
GPLs				
PA	2.49 (−5.70, 10.68)	0.86 (−7.32, 9.04)	6.37 (−1.43, 14.17)	−5.97 (−14.34, 2.40)
PC	2.09 (−6.74, 10.92)	−1.36 (−10.07, 7.35)	0.56 (−8.85, 9.96)	−5.65 (−13.56, 2.26)
PE	−5.09 (−13.87, 3.70)	−3.10 (−11.93, 5.74)	−2.32 (−11.81, 7.18)	−7.86 (−17.02, 1.30)
PG	−2.20 (−10.51, 6.11)	−4.01 (−12.38, 4.36)	5.07 (−3.33, 13.47)	−5.03 (−14.75, 4.69)
PI	1.85 (−6.32, 10.02)	4.68 (−3.56, 12.93)	8.86 (0.38, 17.34)^1^	−0.70 (−9.00, 7.59)
PS	−5.78 (−14.14, 2.57)	−1.38 (−9.80, 7.05)	5.26 (−3.14, 13.66)	−0.06 (−9.96, 9.83)
Lyso-PLs				
LPC	9.93 (1.89, 17.97)^1^	1.42 (−7.31, 10.16)	1.41 (−7.82, 10.65)	−5.86 (−13.97, 2.25)
LPE	−3.71 (−12.76, 5.35)	0.78 (−7.11, 8.66)	1.85 (−6.99, 10.69)	−1.72 (−10.80, 7.35)
LPS	−4.15 (−12.90, 4.59)	2.68 (−5.25, 10.61)	1.81 (−6.51, 10.14)	1.84 (−6.21, 9.90)
Sphingolipids				
Cer	8.24 (0.10, 16.38)^1^	4.44 (−3.60, 12.47)	7.05 (−1.36, 15.46)	−1.65 (−9.86, 6.55)
HexCer	1.11 (−7.55, 9.77)	0.02 (−8.09, 8.13)	3.80 (−4.10, 11.69)	−5.23 (−13.19, 2.72)
SM	−4.09 (−12.68, 4.51)	−1.31 (−9.19, 6.56)	2.23 (−6.58, 11.05)	−3.05 (−11.54, 5.45)
Total	0.12 (−8.45, 8.70)	−0.42 (−8.51, 7.67)	5.66 (−2.67, 14.00)	−5.63 (−13.75, 2.50)

Data are presented as β coefficients (95% confidence intervals) derived from multivariable linear regression models. Models were adjusted for maternal age, pre-pregnancy BMI, parity, mode of delivery, feeding mode, passive smoking, and infant sex. ^1^P < 0.05. Cer, ceramide; GPLs, glycerophospholipids; HexCer, hexosylceramide; LPC, lysophosphatidylcholine; LPE, lysophosphatidylethanolamine; LPS, lysophosphatidylserine; Lyso-PLs, lysophospholipids; PA, phosphatidic acid; PC, phosphatidylcholine; PE, phosphatidylethanolamine; PG, phosphatidylglycerol; PI, phosphatidylinositol; PLs, phospholipids; PS, phosphatidylserine; SM, sphingomyelin; TPL, total phospholipid.

### 3.4 Joint associations between human milk PLs with infant neurodevelopment

To further investigate the overall influence of human milk PLs on infant neurodevelopment, correlations among the 12 PL subclasses were first examined across different lactation stages. The results showed that PLs were generally highly correlated with each other (|r| > 0.5, *p* < 0.05) in all lactation phases, with several correlation coefficients exceeding 0.8, suggesting that single PL indicators may not adequately capture overall PL exposure in human milk. Detailed results are presented in Supplementary Figure S1.

**Figure 2:**
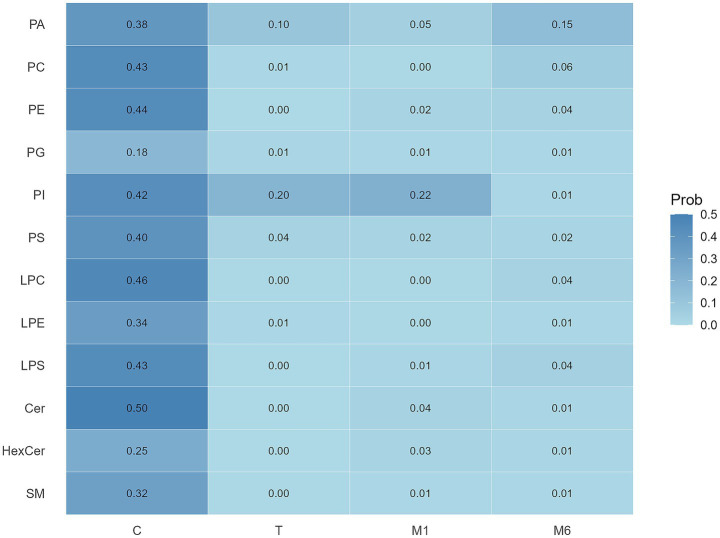
Heatmap of breast milk PLs selection probabilities across lactation stages.

Given the substantial correlations among PLs, Elastic Net regression with bootstrap resampling was applied separately at each lactation stage to evaluate the relative importance and stability of individual phospholipids. As shown in Figure 2, several PL subclasses in colostrum exhibited relatively higher and more stable selection probabilities, including Cer, LPC, PE, PC, LPS, and PI, suggesting their potential joint contribution to infant neurodevelopment at 6 months. In contrast, PLs from later lactation stages generally showed low and unstable selection probabilities, indicating limited and inconsistent contributions.

Multivariable linear regression models were then constructed to evaluate the joint associations between colostrum PLs and infant neurodevelopment at 6 months. The six PL classes identified by Elastic Net were included as independent variables, with adjustment for maternal age, pre-pregnancy BMI, parity, delivery mode, feeding mode, passive smoking exposure, and infant sex. The overall model was statistically significant (*F* = 3.30, *p* = 0.003), and variance inflation factors were low (mean VIF = 1.28), indicating no substantial multicollinearity.

Under joint exposure, higher concentrations of Cer (*β* = 76.94, *p* = 0.008), LPC (*β* = 3.15, *p* = 0.006), and PC (*β* = 0.49, *p* = 0.016) in colostrum were positively associated with neurodevelopment scores at 6 months, whereas PE was negatively associated (*β* = −4.63, *p* < 0.001). PI and LPS were not significantly associated with neurodevelopment (*p* > 0.05). Detailed results are shown in Table 4.

**Table 4:** Joint associations between human milk PLs with infant neurodevelopment: multivariable linear regression models (*n* = 46).

PLs	β	SE	*t* value	*p* value
Cer	76.94	27.33	2.82	0.008
LPC	3.15	1.07	2.94	0.006
PE	−4.63	1.20	−3.85	<0.001
PC	0.49	0.19	2.56	0.016
LPS	−3.36	2.49	−1.35	0.187
PI	0.94	1.46	0.65	0.521

Data are presented as β coefficients (95% confidence intervals) derived from multivariable linear regression models. Models were adjusted for maternal age, pre-pregnancy BMI, parity, mode of delivery, feeding mode, passive smoking, and infant sex. Cer, ceramide; LPC, lysophosphatidylcholine; LPS, lysophosphatidylserine; PC, phosphatidylcholine; PE, phosphatidylethanolamine; PI, phosphatidylinositol; PLs, phospholipids.

To assess the robustness of these findings, sensitivity analyses were conducted by additionally including phospholipids with moderate selection probabilities (PS and PA). The overall pattern of associations remained largely consistent, with no material changes in the direction or statistical significance of the main results. Detailed results are presented in Supplementary Table S1.

## 4 Discussion

In this prospective cohort study, we systematically analyzed the dynamic changes of breast milk PL concentrations across different lactation stages and investigated their associations with infant neurodevelopment at 6 months of age. We observed pronounced temporal variations in breast milk PLs during lactation, with TPL concentrations decreasing from colostrum to mature milk and stabilizing thereafter. Different PL subclasses displayed distinct dynamic patterns. In multivariable linear regression models, LPC and Cer in colostrum, as well as PI in 1-month mature milk, were positively associated with infant neurodevelopment scores at 6 months. Considering correlations among PLs, Elastic Net regression identified key PL components in colostrum associated with neurodevelopment. In the joint model, Cer, LPC, and PC were positively associated, whereas PE was negatively associated with infant neurodevelopment. Overall, these findings suggest that PLs in early lactation milk may play an important role in infant neurodevelopment.

We observed marked dynamic changes in breast milk PLs across lactation stages, which are generally consistent with previous studies. TPL concentrations decreased from colostrum to mature milk and then stabilized, a pattern also reported in large Chinese breast milk cohorts (22–24). Prior studies have indicated that breast milk PL composition is not static but dynamically adjusted according to lactation stage and maternal metabolic status (21, 25). Specifically, in our study, PC, PG, LPS, and multiple sphingolipids gradually decreased overg lactation, suggesting that different PL classes may fulfill stage-specific physiological roles during early life.

Regarding the associations with infant neurodevelopment, we found that LPC and Cer in colostrum were positively associated with neurodevelopment scores at 6 months. LPC is an important intermediate in PL metabolism and is considered a major carrier of long-chain polyunsaturated fatty acids into the brain, particularly docosahexaenoic acid (DHA) (26). Studies have shown that the blood–brain barrier transporter Mfsd2a specifically transports DHA in the form of LPC, facilitating DHA accumulation in brain tissue and supporting neuronal membrane formation, synaptogenesis, and myelination (27–29). Variations in breast milk LPC concentrations may therefore influence DHA availability in the infant brain and subsequently affect early neurodevelopment. Cer, a central hub in sphingolipid metabolism, is a key structural component of neuronal membranes and myelin (30). Previous studies have demonstrated that Cer participates in membrane maintenance, neuronal differentiation, and signal transduction during neural development (31). As an important metabolic intermediate, Cer can also be converted to sphingomyelin and hexosylceramides, which are critical for membrane stability and myelination (32). Higher levels of Cer in breast milk may therefore provide essential structural lipids that support early brain development.

In addition, PI in 1-month mature milk was positively associated with infant neurodevelopment. PI and its derivatives serve as key signaling lipids in cell membranes and regulate multiple intracellular pathways, including calcium signaling, membrane trafficking, and protein kinase activation (33, 34). These signaling processes are involved in cell proliferation, differentiation, and migration, all of which are essential for neural development and neuronal function. Thus, PI may indirectly support early neurodevelopment by maintaining neuronal signaling and membrane dynamics.

Besides LPC and Cer, PC was also positively associated with infant neurodevelopment in the joint model. PC is the most abundant PL in cell membranes and a major dietary source of choline, which is a precursor for acetylcholine and membrane PL synthesis (35). Adequate choline intake is critical for fetal and early infant brain development (36). Therefore, higher PC levels in breast milk may promote neurodevelopment by providing both structural membrane components and substrates for neurotransmitter synthesis.

Conversely, PE showed a negative association with infant neurodevelopment in the joint model, which should be interpreted cautiously. PE is an important membrane phospholipid involved in maintaining membrane curvature, protein function, and mitochondrial membrane integrity (37). However, PE is metabolically interconnected with other PLs and participates in multiple remodeling pathways that maintain phospholipid homeostasis (38, 39). In our correlation analyses, strong associations were observed among PL subclasses in colostrum, particularly between PE and major membrane PLs such as PC and PG (r > 0.5, *p* < 0.001). This suggests that PL concentrations in breast milk may change in a coordinated manner through shared metabolic pathways, such as the Lands cycle. Therefore, the negative association observed for PE may not represent an independent biological effect but rather reflect shifts in relative PL composition when other PLs are considered simultaneously. For instance, alterations in the PE/PC ratio could influence membrane structural properties and lipid metabolism, thereby indirectly affecting neurodevelopmental processes. Previous studies have highlighted the importance of maintaining the balance between PC and PE for cellular function, organ health, and systemic metabolism, with imbalances linked to various pathological conditions (40). Future studies are warranted to explore whether the balance among major PL classes, such as the PC/PE ratio, contributes to early neurodevelopment.

Moreover, the overall PE concentrations in our cohort were lower than reported in some previous populations, which may relate to differences in maternal nutritional status, metabolic factors, lifestyle, or other cohort characteristics (22, 23). The relatively small sample size of our study may also influence the distribution of PL concentrations and their associations with neurodevelopment, underscoring the need for validation in larger and more diverse populations.

Notably, the associations between breast milk PLs and infant neurodevelopment in our study were primarily observed for colostrum, whereas no significant direct associations were detected for 6-month mature milk. This finding suggests the possibility of a critical exposure window during early lactation, during which breast milk PLs may exert stronger influences on early neural development. However, previous work by Yang et al. reported that breast milk PLs were associated with cognitive development primarily during 200–400 days of lactation (late mature milk stage) (41). Therefore, the absence of significant associations at 6 months in our study does not exclude potential longer-term effects. Longer follow-up, such as assessments at 12 months or later, may help clarify the sustained impact of breast milk PLs on neurodevelopment.

Beyond biological implications, our findings may provide preliminary insights for early-life nutritional applications. In particular, phospholipid classes such as LPC, PC, and sphingolipids associated with infant neurodevelopment may inform future efforts to optimize the lipid composition of infant formula. However, further mechanistic and clinical studies are required before translational application.

This study has several strengths. First, the prospective mother–infant cohort design enabled longitudinal assessment of breast milk PLs across multiple lactation stages. Second, UPLC–MS/MS was used to quantify a broad range of breast milk PLs, providing high analytical sensitivity and reliability. Third, the combined use of multivariable regression and Elastic Net approaches helped address potential multicollinearity among PLs, thereby improving the robustness of the findings.

Several limitations should also be considered. First, the relatively small sample size may limit statistical power and the stability of some associations. Second, infant neurodevelopment was assessed only at 6 months, which may not fully capture longer-term cognitive outcomes. Third, although multiple potential confounders were adjusted for, residual confounding cannot be excluded. Importantly, given that a substantial proportion of infants received mixed feeding, including infant formula and complementary foods, the estimated phospholipid exposure primarily reflects breast milk derived lipid concentrations. Therefore, the exposure variable should be interpreted as a proxy of breast milk lipid composition within a real-world mixed feeding context, and the findings may not fully represent the biological effects of exclusive breastfeeding or total dietary phospholipid exposure in early infancy. Fourth, the selection probabilities of phospholipids were generally modest, reflecting limited stability in variable selection, which may also be related to the small sample size. To address this, sensitivity analyses including additional phospholipids (PS and PA) were performed, and the main associations remained largely consistent, supporting the robustness of our findings. Finally, our analysis focused on PL subclasses rather than individual molecular species, which may differ in fatty acid composition and biological function. Future studies should therefore investigate breast milk PLs at the molecular species level to better elucidate their roles in infant neurodevelopment.

## Data Availability

The original contributions presented in the study are included in the article/Supplementary material, further inquiries can be directed to the corresponding authors.
