# Supplementary figures and images for: Human milk phospholipids across lactation stages and their associations with infant neurodevelopment: a prospective cohort study in China
Source: Front Nutr. 2026 May 21;13:1841752. doi: 10.3389/fnut.2026.1841752 (PMC13233376; doi:10.3389/fnut.2026.1841752)

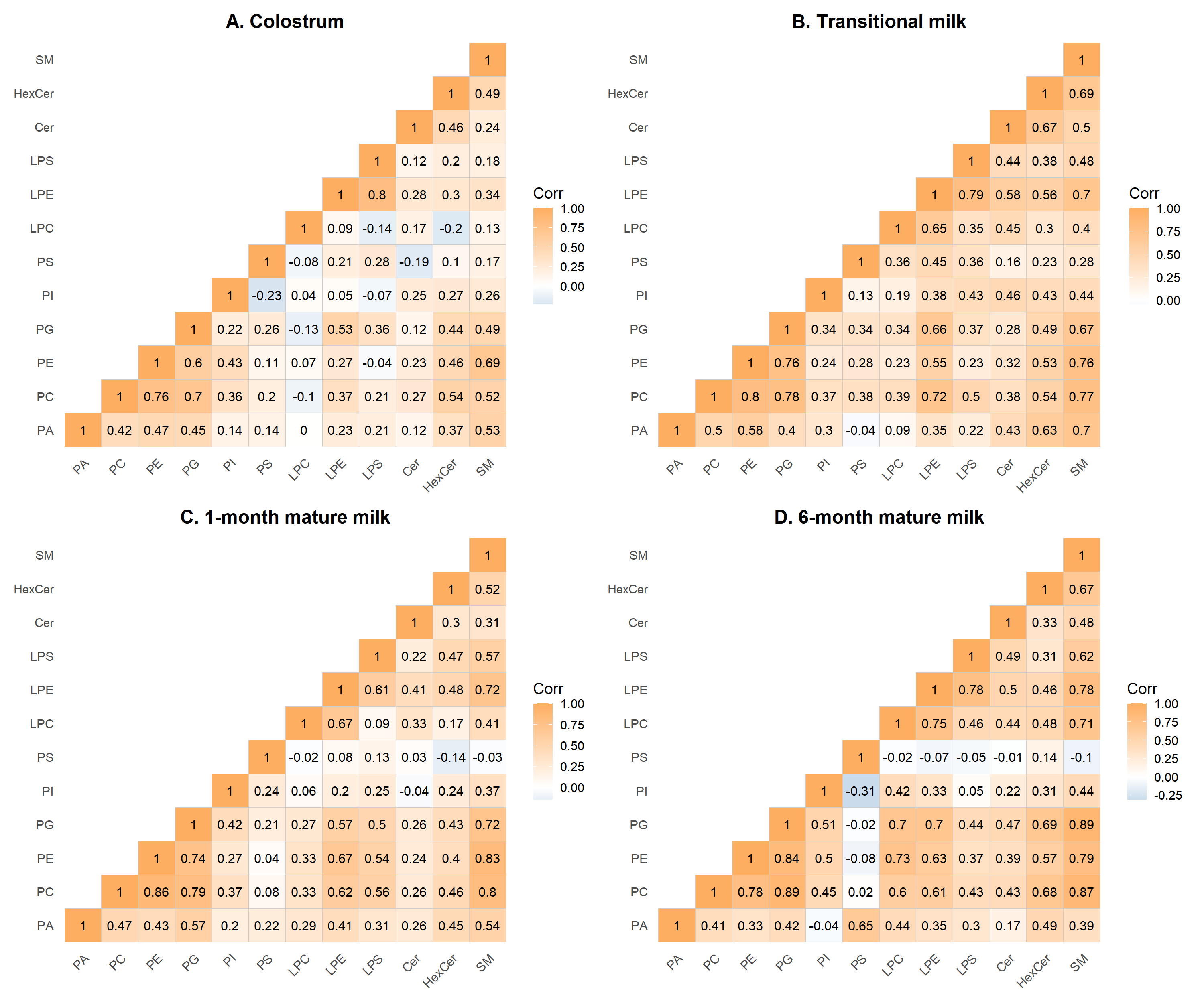

Supplement: Supplementary file 1 [file Image_1.JPEG]
