# Supplementary material for: Human milk phospholipids across lactation stages and their associations with infant neurodevelopment: a prospective cohort study in China
Source: Front Nutr. 2026 May 21;13:1841752. doi: 10.3389/fnut.2026.1841752 (PMC13233376; doi:10.3389/fnut.2026.1841752)
Supplement: Supplementary file 2 [file Table_1.DOCX]

Supplementary Material

**Supplementary Table S1**. Pairwise comparisons of breast milk phospholipids across lactation stages using Nemenyi test (n = 50).

| **PLs** | **C vs T** | **C vs M1** | **C vs M6** | **T vs M1** | **T vs M6** | **M1 vs M6** |
| --- | --- | --- | --- | --- | --- | --- |
| PC | 0.042 | <0.001 | <0.001 | 0.010 | <0.001 | 0.006 |
| PG | 0.093 | <0.001 | <0.001 | 0.052 | <0.001 | 0.010 |
| PI | 0.093 | 0.455 | 0.002 | 0.001 | <0.001 | 0.132 |
| PS | 0.093 | 0.829 | 0.213 | 0.455 | <0.001 | 0.027 |
| LPC | <0.001 | <0.001 | <0.001 | 0.996 | 0.926 | 0.829 |
| LPE | 0.034 | 0.052 | 0.246 | 0.999 | 0.829 | 0.898 |
| LPS | <0.001 | <0.001 | <0.001 | 0.999 | 0.034 | 0.052 |
| Cer | 1.000 | <0.001 | <0.001 | <0.001 | <0.001 | 0.321 |
| HexCer | 0.503 | <0.001 | <0.001 | 0.052 | <0.001 | 0.063 |
| SM | 1.000 | 0.005 | <0.001 | 0.005 | <0.001 | 0.321 |
| TPL | 0.602 | 0.034 | 0.022 | 0.455 | 0.364 | 0.999 |

Abbreviations: C, colostrum; T, transitional milk; M1, 1-month mature milk; M6, 6-month mature milk; Cer, ceramide; HexCer, hexosylceramide; LPC, lysophosphatidylcholine; LPE, lysophosphatidylethanolamine; LPS, lysophosphatidylserine; PC, phosphatidylcholine; PG, phosphatidylglycerol; PI, phosphatidylinositol; PLs, phospholipids; PS, phosphatidylserine; SM, sphingomyelin; TPL, total phospholipid.


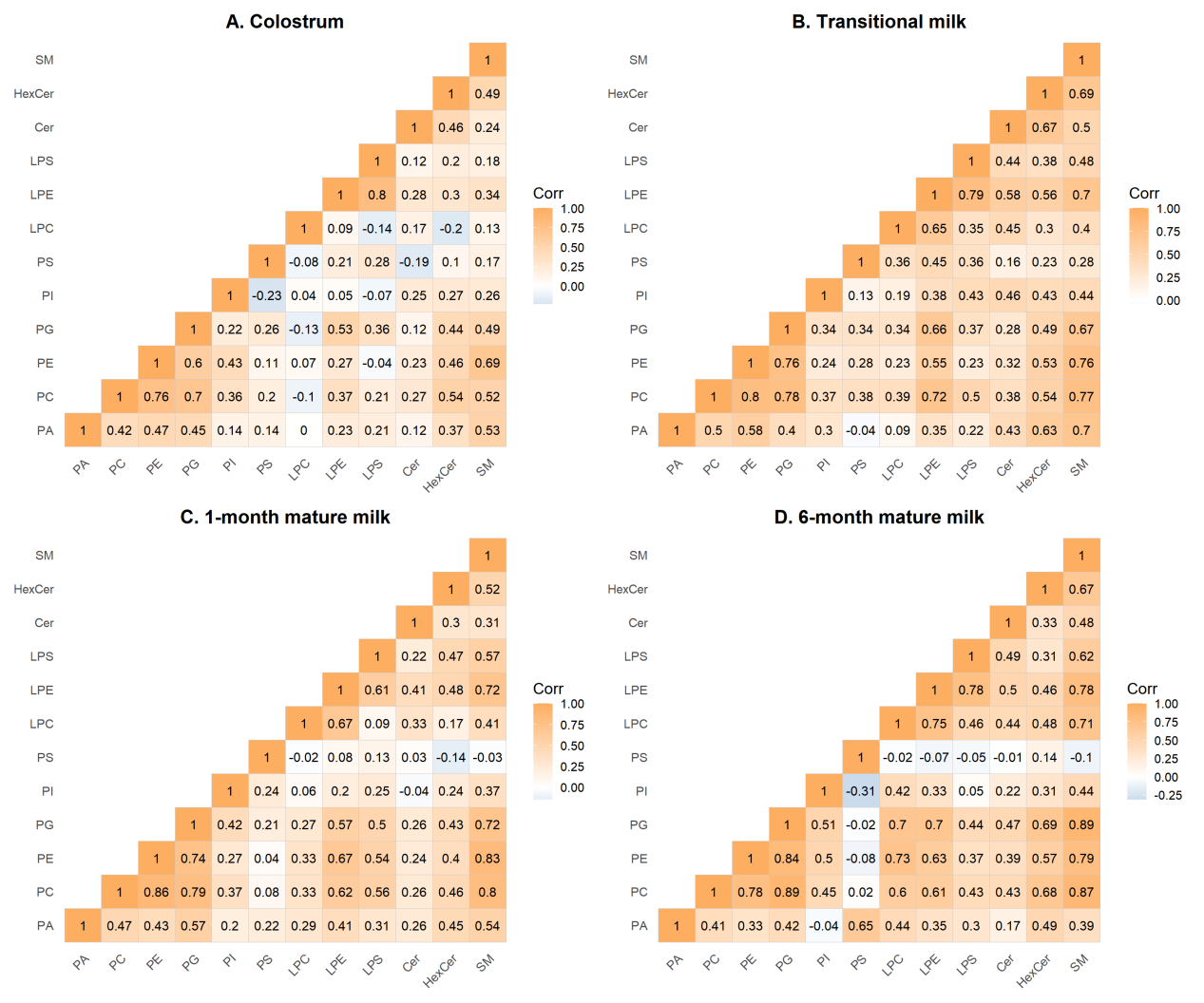


**Supplementary Figure S1.** Spearman correlation matrices of breast milk PL subclasses across lactation stages.

**Supplementary Table S2**. Joint Associations Between Human Milk PLs including additional phospholipids (PS and PA) With Infant Neurodevelopment: multivariable linear regression models (n = 46).

| **PLs** | **β** | **SE** | ***t*** value | ***P*** value |
| --- | --- | --- | --- | --- |
| Cer | 63.09 | 29.4 | 2.15 | 0.040 |
| LPC | 3.08 | 1.09 | 2.82 | 0.008 |
| PE | -4.77 | 1.21 | -3.93 | <0.001 |
| PC | 0.49 | 0.2 | 2.40 | 0.023 |
| LPS | -3.62 | 2.71 | -1.34 | 0.191 |
| PI | 1.14 | 1.49 | 0.77 | 0.450 |
| PS | -0.52 | 0.46 | -1.13 | 0.270 |
| PA | 0.08 | 0.08 | 0.94 | 0.353 |

Data are presented as β coefficients (95% confidence intervals) derived from multivariable linear regression models. Models were adjusted for maternal age, pre-pregnancy BMI, parity, mode of delivery, feeding mode, passive smoking, and infant sex. Abbreviations: Cer, ceramide; LPC, lysophosphatidylcholine; LPS, lysophosphatidylserine; PA, phosphatidic acid; PC, phosphatidylcholine; PE, phosphatidylethanolamine; PI, phosphatidylinositol; PLs, phospholipids; PS, phosphatidylserine.
